# Supplementary figures and images for: Allele surfing causes maladaptation in a Pacific salmon of conservation concern
Source: PLoS Genet. 2023 Sep 8;19(9):e1010918. doi: 10.1371/journal.pgen.1010918 (PMC10545117; doi:10.1371/journal.pgen.1010918)

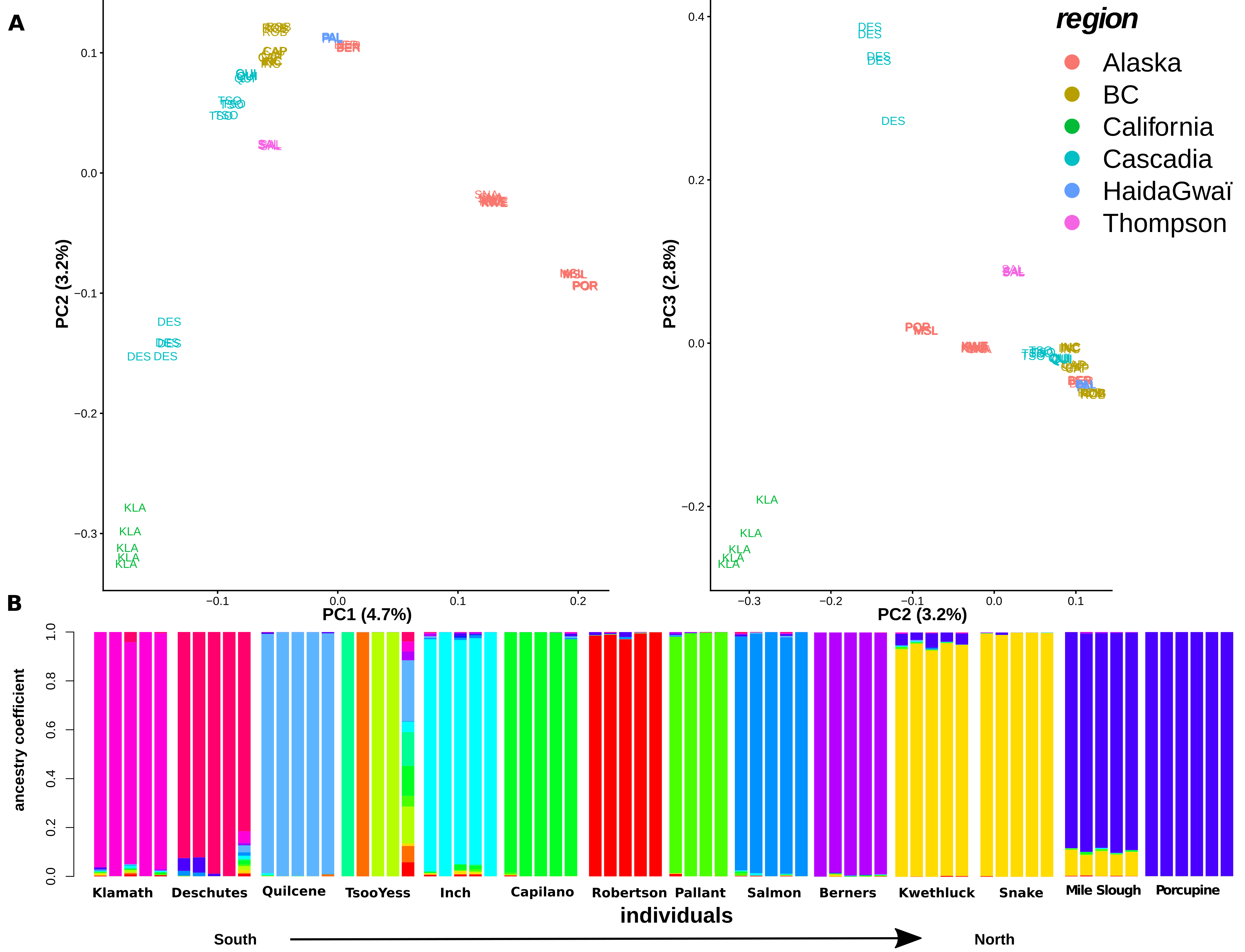

Supplement: S1 Fig — A. Result of a principal component analysis obtained from a set of high quality biallelic SNPs without missing data showing both a clusterization along latitude and longitude as well as discrete clusters corresponding broadly to each river. Each label represents a given individual from a given river (labelled following table S01) and is coloured according to its region of sampling. B. LEA results for admixture inference for K = 14. Each bar represents an individual and is coloured according to its membership probability. Each name corresponds to a river. The TsooYes appear as a mixture of different individuals. Results must be interpreted with caution given the small sample sizes. (TIF) [file pgen.1010918.s013.tif]

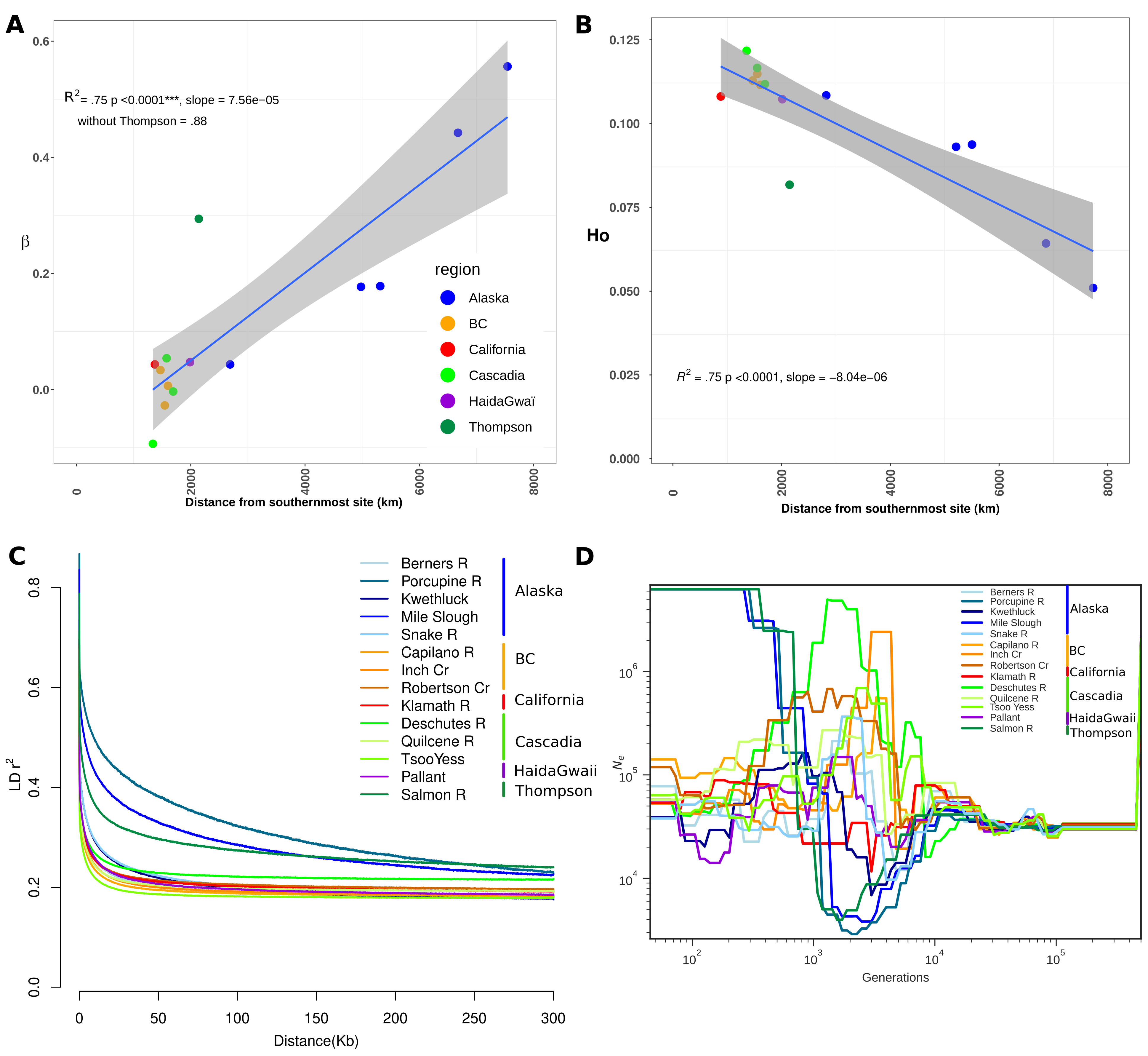

Supplement: S2 Fig — A. Positive correlation between the βST and distance to the southernmost site showing that differentiation increased linearly from the south to the north. In all panels each point represents a sampling site and is coloured according to the region in which it was sampled. The most negative values display likely ancestral samples. The Thompson sample displays high inbreeding and is bottlenecked. Displayed is the adjusted R2 of a linear model along with its p-value. The grey area represents the 95% confidence interval levels around the regression lines obtained with the predict function in R. B. Negative correlation between genetic diversity (observed heterozygosity) and distance to the south.The Thompson sample displays high inbreeding and is bottlenecked. Displayed is the adjusted R2 of a linear model along with its p-value. The grey area represents the 95% confidence interval levels around the regression lines obtained with the predict function in R. C. Rates of LD decay as a function of distance along the genome. The higher LD indicates a history of inbreeding or bottleneck. D. SMC++ inference of population size change with whole genome sequences for each local population of Coho salmon. Recent times should be interpreted carefully. (TIF) [file pgen.1010918.s014.tif]

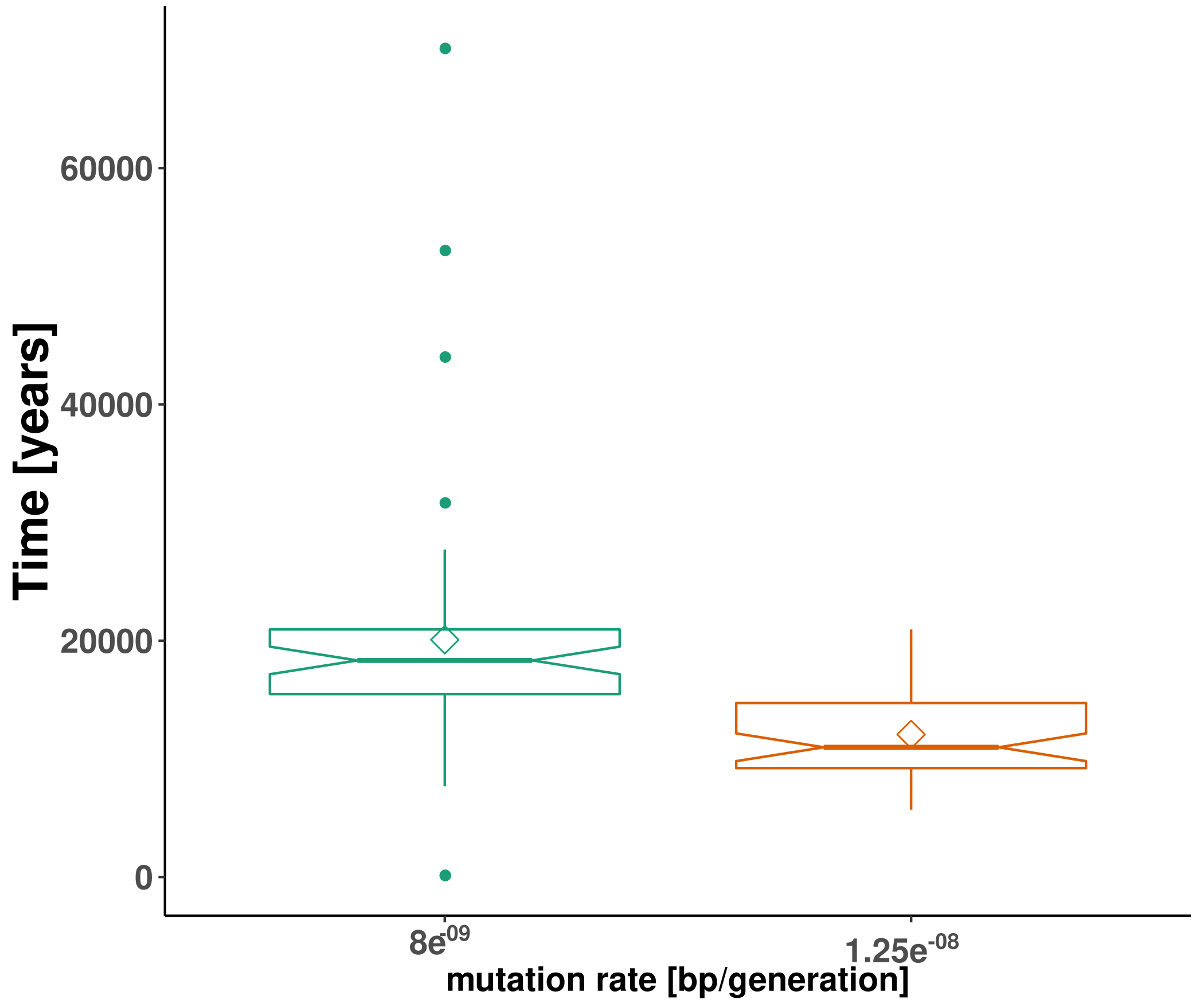

Supplement: S3 Fig — A) Tree with three migration arrows. Each name describes a river sample site. Each river is color coded following the color scheme provided elsewhere (e.g. Fig 1). 3 significant migration arrows are displayed. Each migration arrow is colored according to the weight it received (from yellow to red) in Treemix. The weights are related to the fraction of alleles in the descendant population that originated in each donor population. Each node was highly supported based on 500 bootstrap. B) proportion of variance in the covariance of allele frequency explained as a function of the number of migration edges. C) Same tree colored according to the values of πN/πS (TIF) [file pgen.1010918.s015.tif]

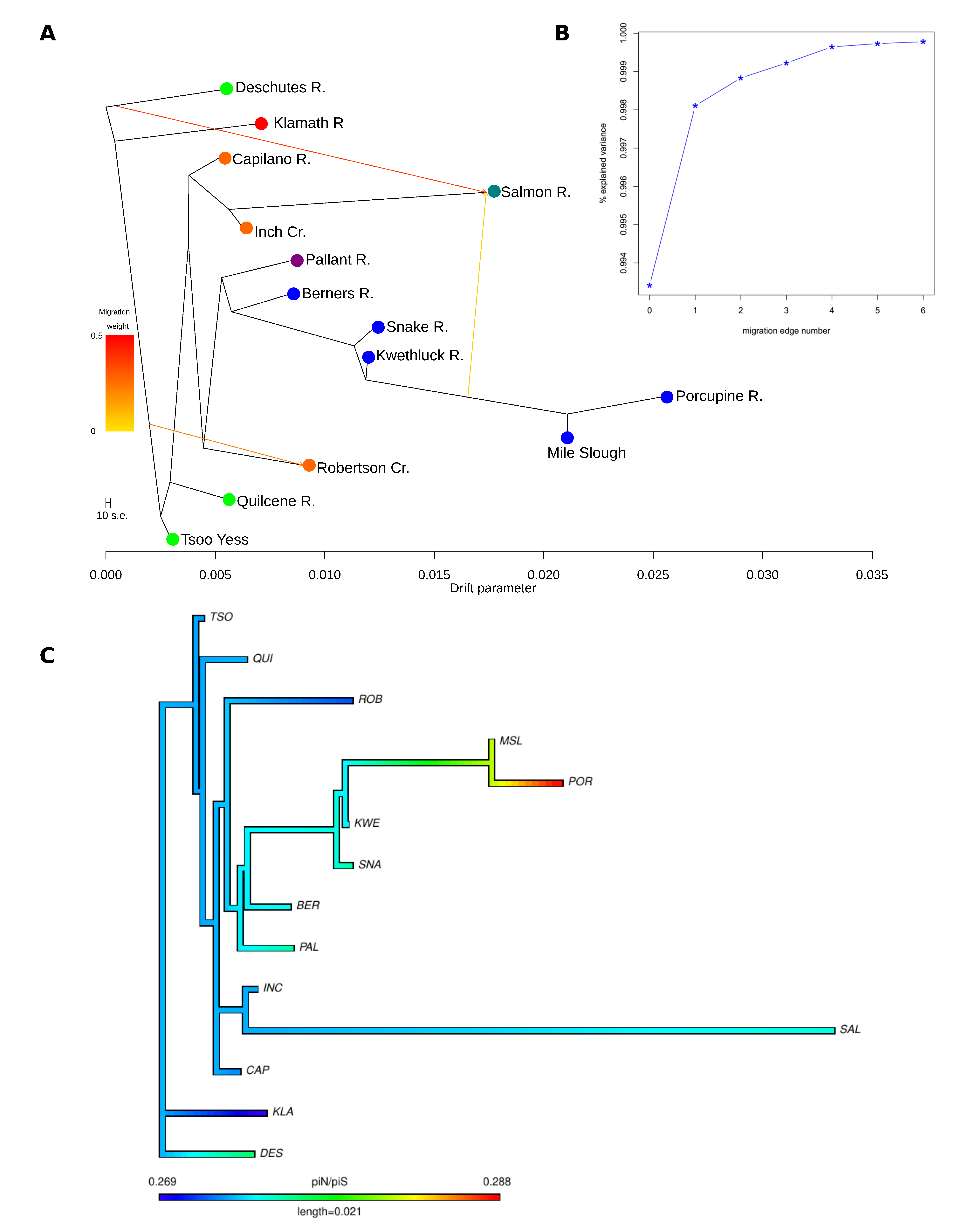

Supplement: S4 Fig — Estimates of population split time from SMC++ under a model without gene flow among populations. Shown are estimates obtained when comparing split time between pairs of samples from different major regional groups. Two different mutation rates were used the: mean and median values based on Salmo salar orthologues mapped on the pike Esox lucius genome (Wang J. personal communication). (TIF) [file pgen.1010918.s016.tif]

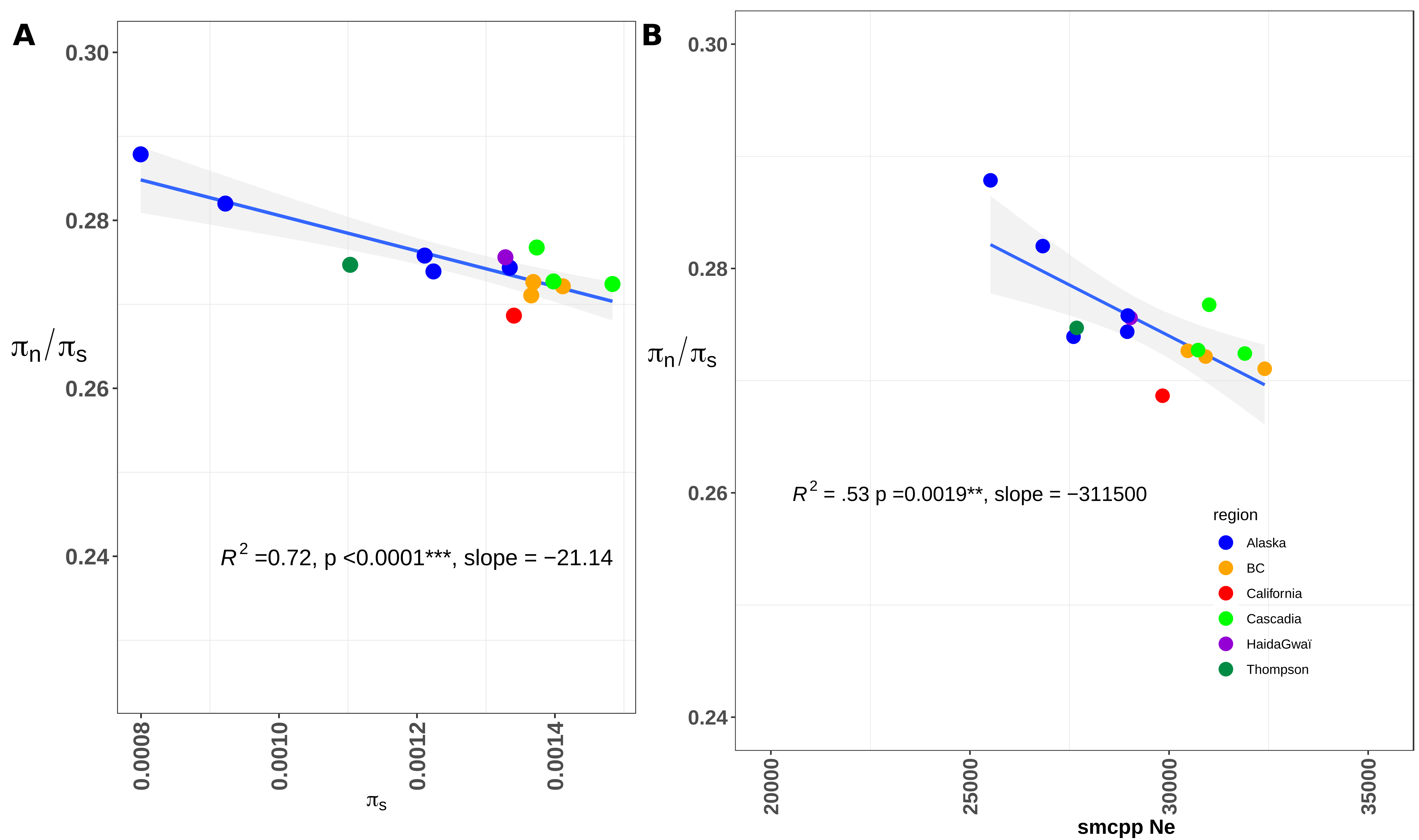

Supplement: S5 Fig — A) Distribution of πN/πS as a function of πS in each coho salmon populations from the study. B) Distribution of πN/πS as a function of Ne from SMC++ for each coho salmon populations from the study. Results of linear models are displayed. In all panels each point represents a sampling site and is coloured according to the region in which it was sampled. Displayed is the adjusted R2 of a linear model along with its p-value. The grey area represents the 95% confidence interval levels around the regression lines obtained with the predict function in R.+ (TIF) [file pgen.1010918.s017.tif]

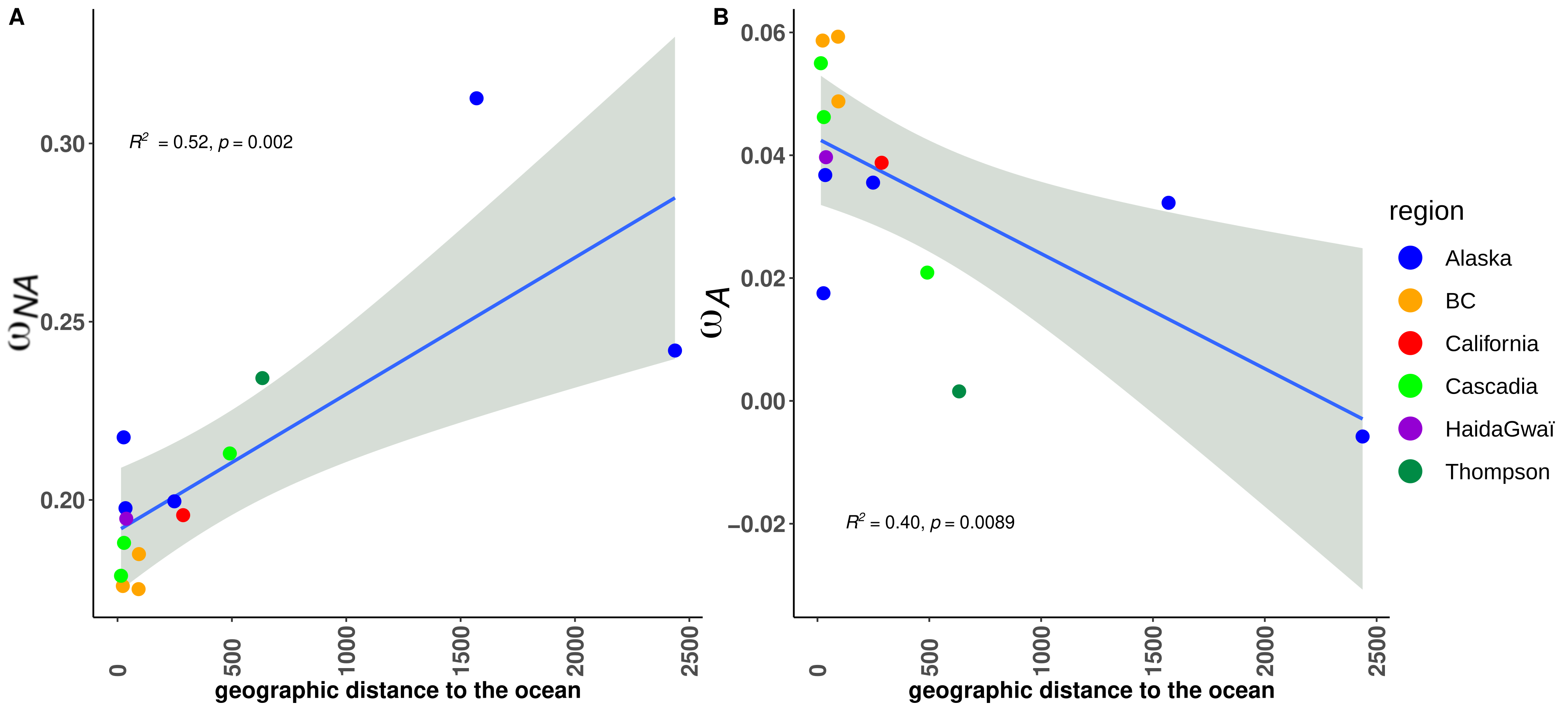

Supplement: S6 Fig — Correlation between distance to the ocean of each sample location (i.e. corresponding to the spawning migration) and the inferred rate of A) non-adaptive substitution (ωNA) and B) adaptive substitution (ωA). In all panels each point represents a sampling site and is coloured according to the region in which it was sampled. Displayed is the adjusted R2 of a linear model along with its p-value. The grey area represents the 95% confidence interval levels around the regression lines obtained with the predict function in R. (TIF) [file pgen.1010918.s018.tif]

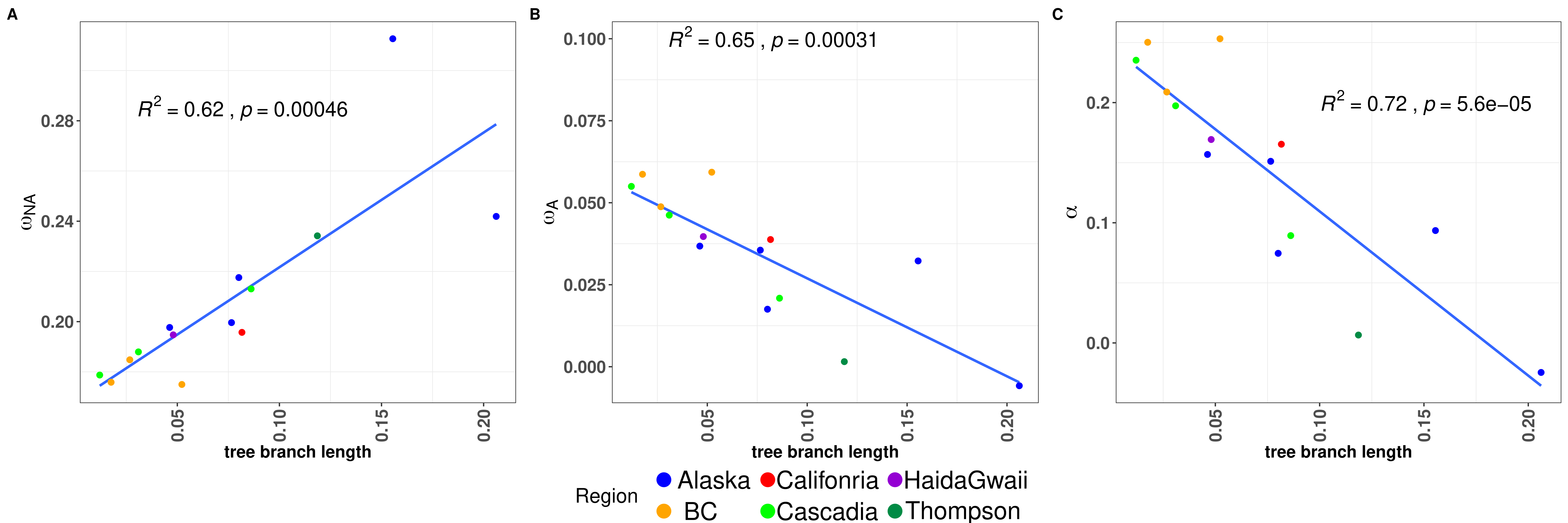

Supplement: S7 Fig — A: relationship between ωNA and tree branch length; B: relationship between ωA and tree branch length; C: relationship between α and tree branch length. See text for a definition of each metrics. Sample sites are coloured by region. The blue line represents the value of the regression line. In all panels each point represents a sampling site and is coloured according to the region in which it was sampled. Displayed is the adjusted R2 along with its p-value. (TIF) [file pgen.1010918.s019.tif]

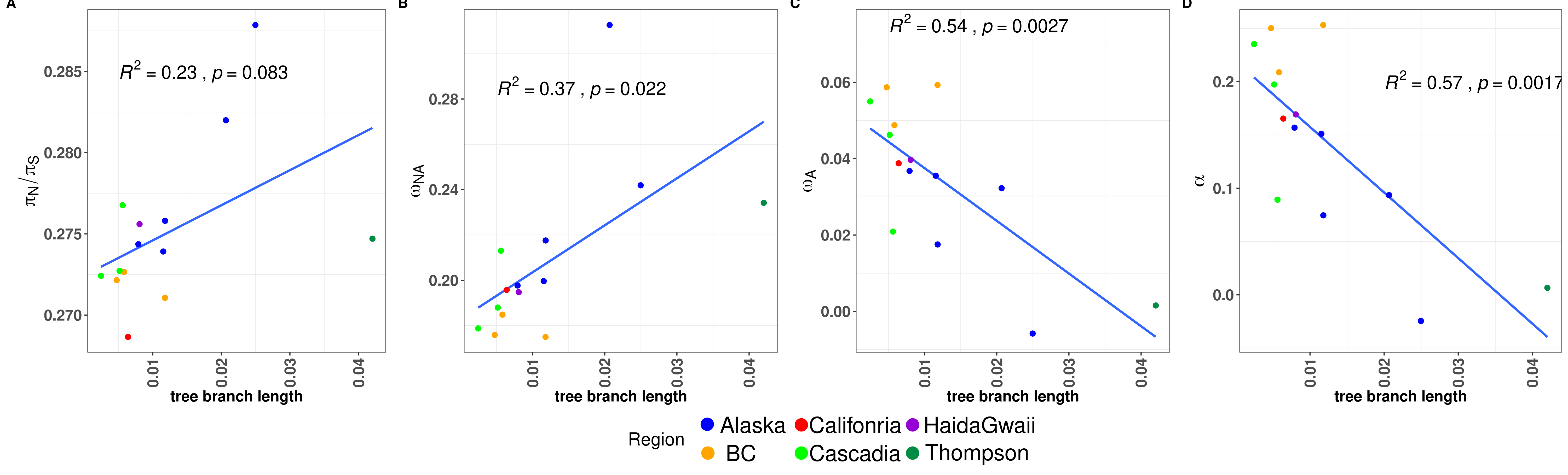

Supplement: S8 Fig — Results of linear models testing the effect of tree branch length to the root extracted from a treemix population phylogeny on the load (πN/πS, panel A) and different metrics of selection efficacy B: relationship between ωNA and tree branch length; C: relationship between ωA and tree branch length; D: relationship between α and tree branch length. See text for a definition of each metrics). Sample sites are coloured by region. The blue line represents the value of the regression line. In all panels each point represents a sampling site and is coloured according to the region in which it was sampled. Displayed is the adjusted R2 along with its p-value. (TIF) [file pgen.1010918.s020.tif]

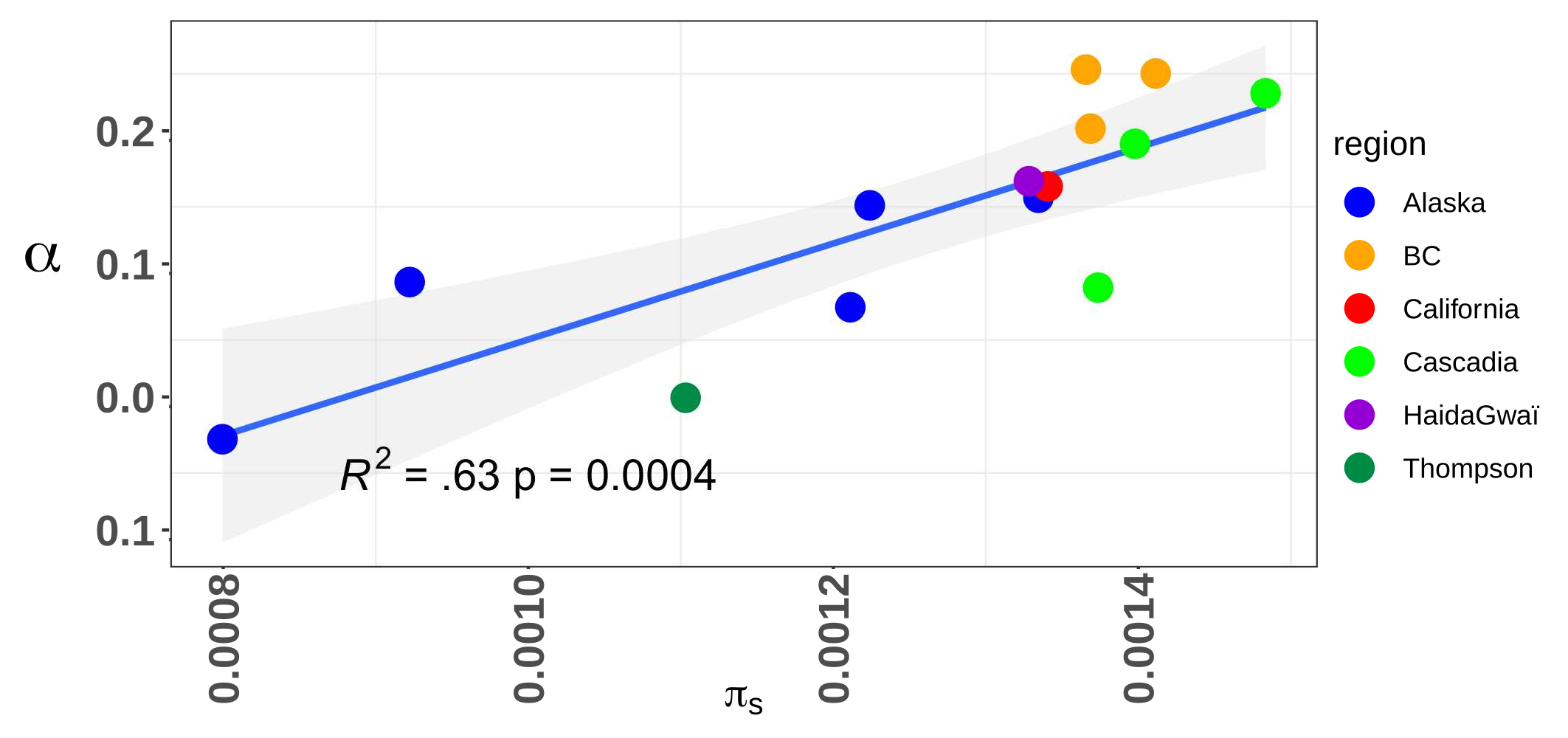

Supplement: S9 Fig — Each point represents a sampling site and is coloured according to the region in which it was sampled. Displayed is the adjusted R2 along with its p-value. The grey area represents the 95% confidence interval levels around the regression lines obtained with the predict function in R. (TIF) [file pgen.1010918.s021.tif]

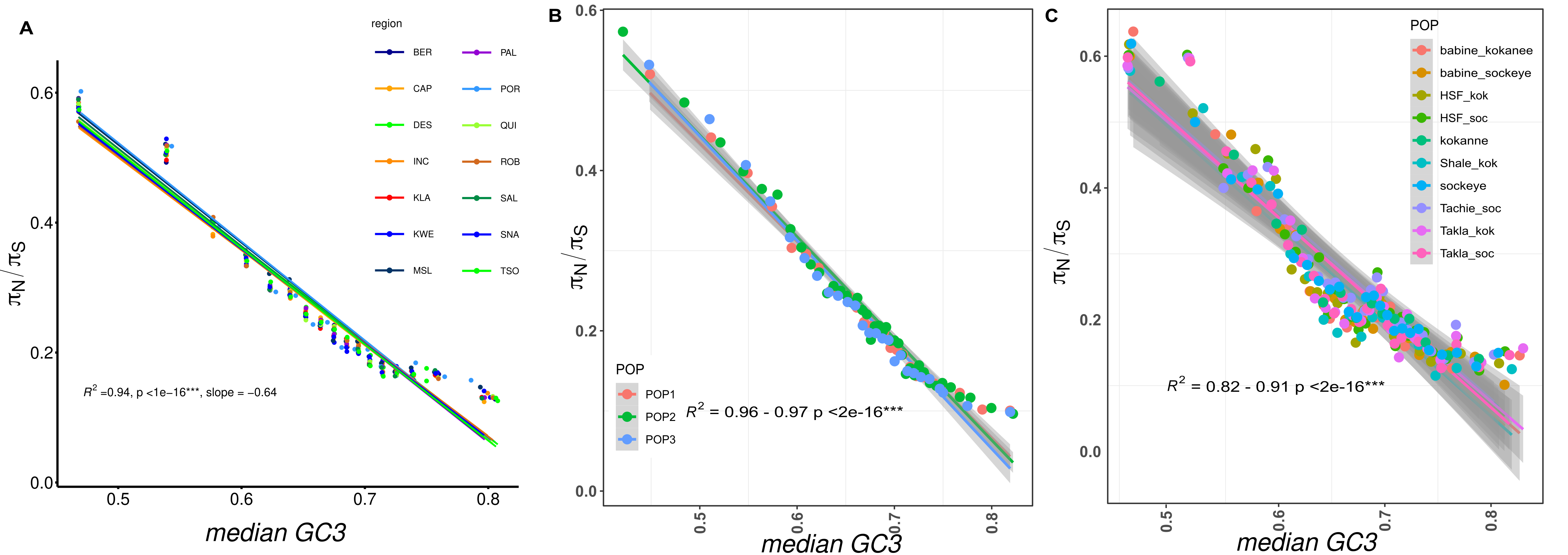

Supplement: S10 Fig — A) Correlation for each population of coho salmon. Each point represents a sampling site and is coloured according to the region in which it was sampled; B) correlation within populations of rainbow trout. Each point represent a population as infered using a PCA and corresponds to different rivers of sampling. C) correlation for each population of Sockeye and Kokanee ecotype. Each point corresponds to differents rivers. All correlations are significant. The x-axis displays the median GC3 and y-axis the πN/πS ratio. Abbreviation for each site is available in Table S01. Displayed is the adjusted R2 of a linear model along with its p-value. The grey area represents the 95% confidence interval levels around the regression lines obtained with the predict function in R. (TIFF) [file pgen.1010918.s022.tiff]

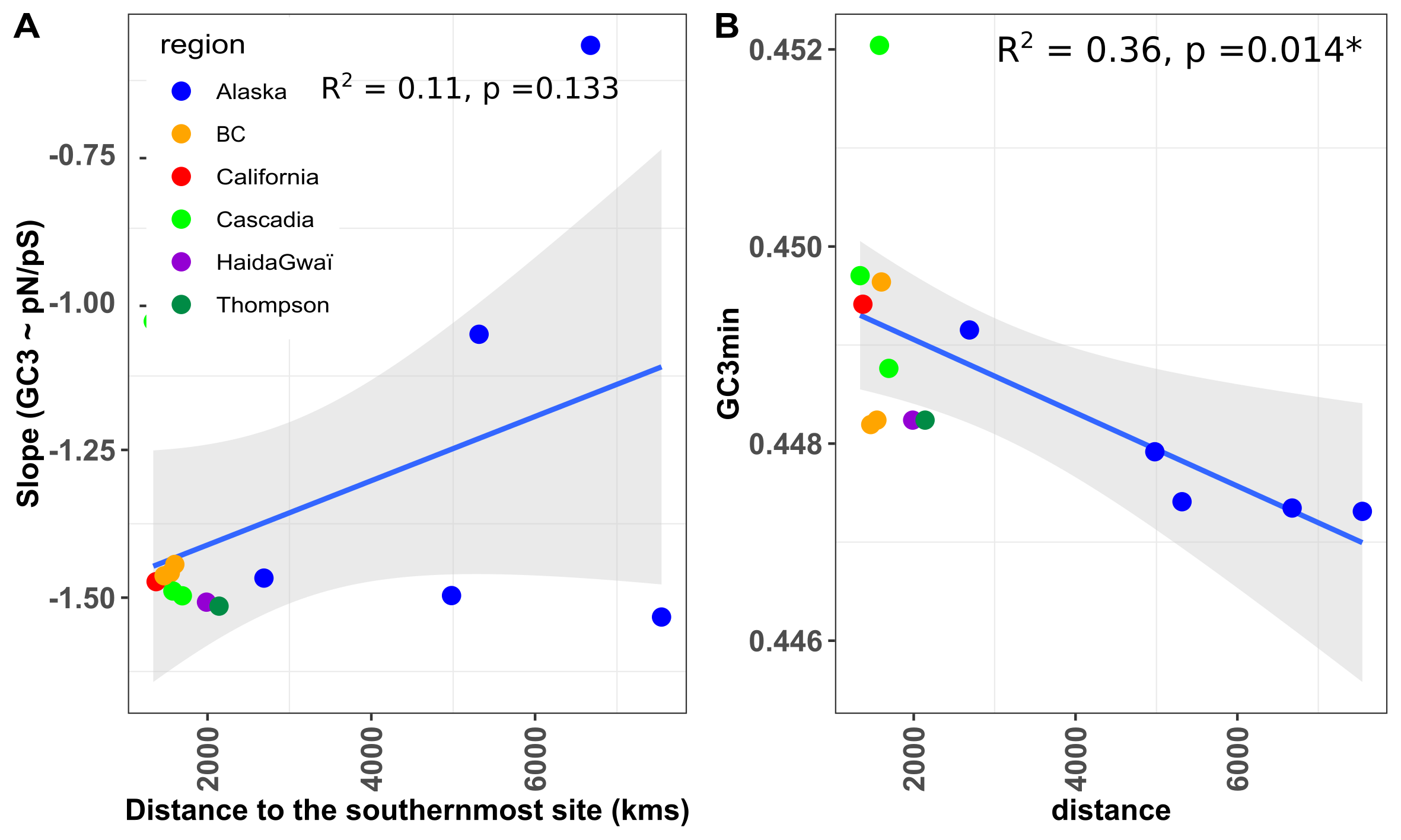

Supplement: S11 Fig — A) relationship between the slope of the linear model between GC3 ~ and πN/πS and the distance to the southernmost site. B) Correlation between the lowest recombining GC3 classes (expected to display the highest load) and the distance to the southernmost sites. In all panels each point represents a sampling site and is coloured according to the region in which it was sampled. Displayed is the adjusted R2 of a linear model along with its p-value. The grey area represents the 95% confidence interval levels around the regression lines obtained with the predict function in R. (TIFF) [file pgen.1010918.s023.tiff]

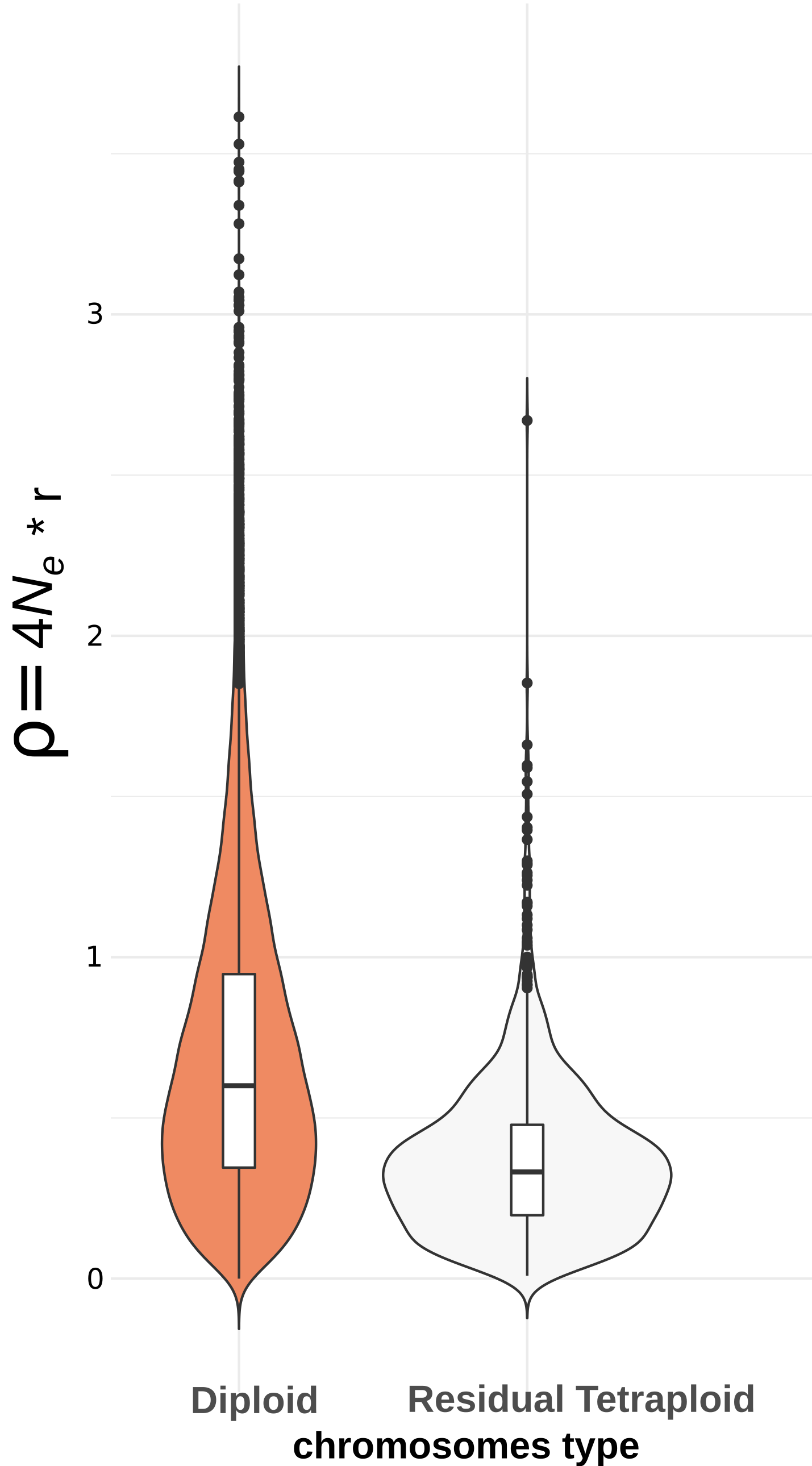

Supplement: S12 Fig — Combined Violin plot and boxplot showing the distribution of population scale recombination (⍴ = 4*Ne*r) inferred from LDhat in Diploid chromosomes (orange) vs the 8 Regions of residual tetraploidy (gray). (TIF) [file pgen.1010918.s024.tif]

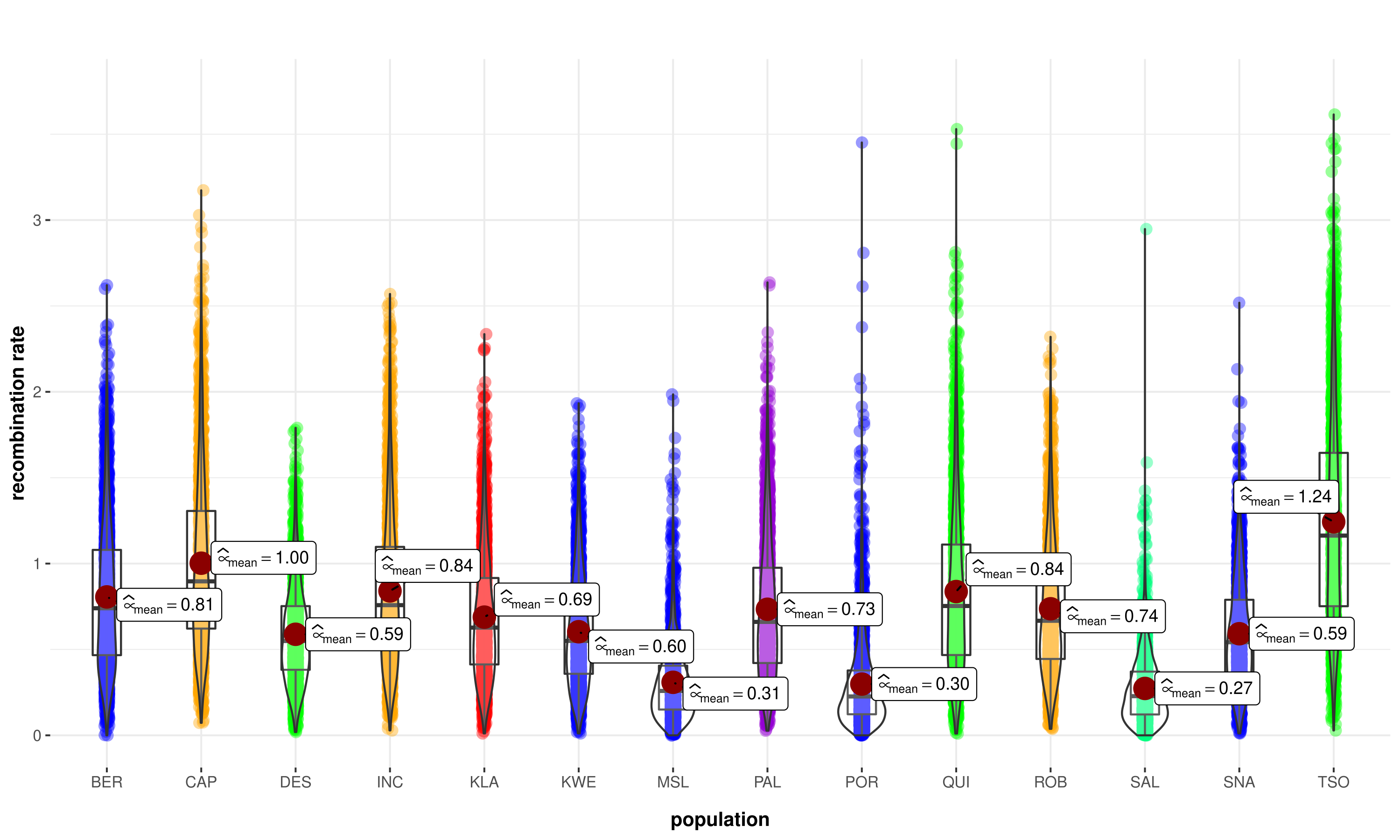

Supplement: S13 Fig — Each point represents the observed value of population scale recombination rate (ρ = 4Ne*μ) computed in 1 mb windows over the whole genome in each population. The harmonic mean is plotted by a red dot along with its value. For each population a violin plot embedded within a boxplot is shown. (TIF) [file pgen.1010918.s025.tif]

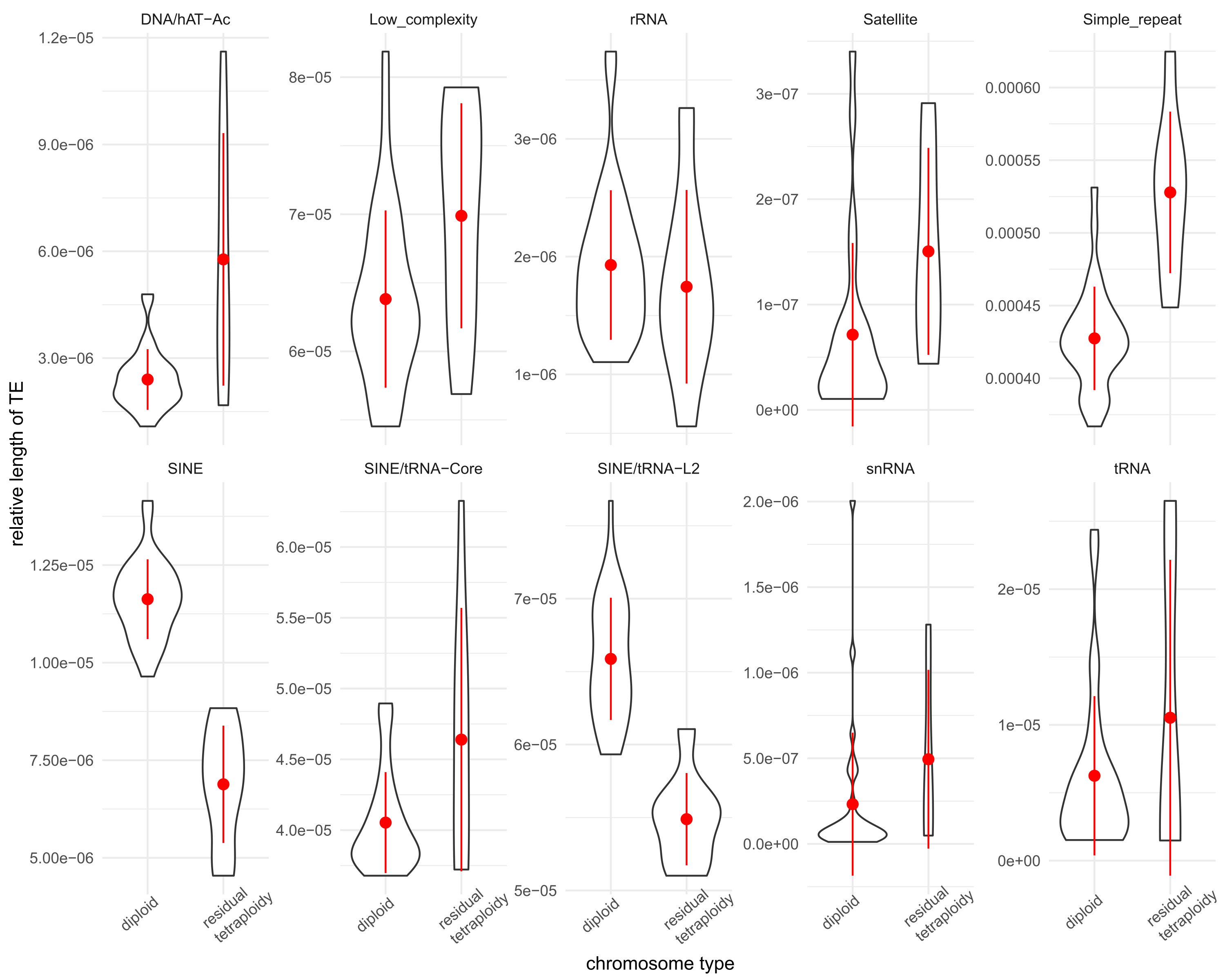

Supplement: S14 Fig — Violin plot displaying the difference in TEs relative length (i.e. length corrected by the total length of each chromosome) for each major TE category and each type of chromosome. Red point = mean +/- 1*sd. (TIFF) [file pgen.1010918.s026.tiff]

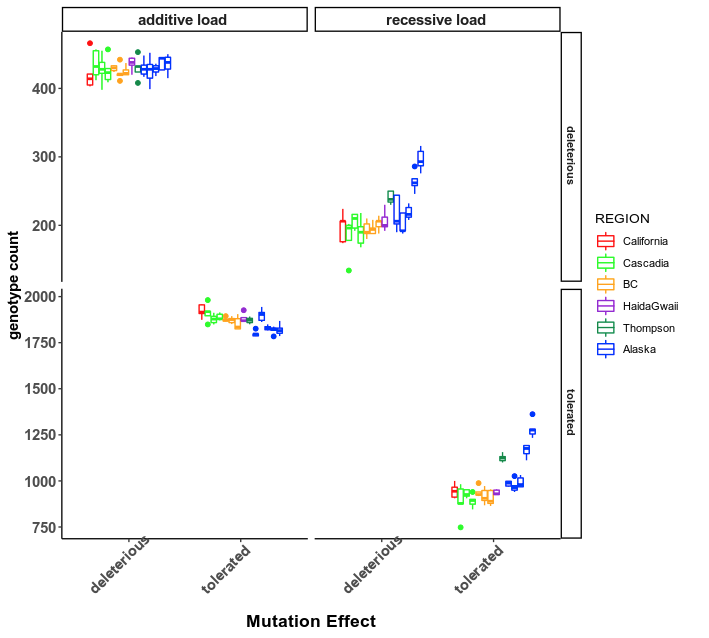

Supplement: S15 Fig — Boxplot showing the number of deleterious alleles per river (sorted from the south to to north). left panel = additive load, right panel = recessive load. top = missense deleterious mutations according to Provean predictions, bottom = missense tolerated mutations according to Provean predictions. No strong differences are observed in the additive load among populations. Significant differences were observed for the recessive load in populations at the expansion front which is qualitatively similar to our inferences from missense and LoF mutations. Each color represents a major regional group. Results were obtained for a random subset of mutations only given the strong computational burden of Provean. (TIF) [file pgen.1010918.s027.tif]
