# Supplementary material for: Allele surfing causes maladaptation in a Pacific salmon of conservation concern
Source: PLoS Genet. 2023 Sep 8;19(9):e1010918. doi: 10.1371/journal.pgen.1010918 (PMC10545117; doi:10.1371/journal.pgen.1010918)
Supplement: Supplementary file 14 [file pgen.1010918.s010.odt]

Supplementary Note S1: Reconstruction of demographic history from RADseq data

Previous work [1] based on ∂a∂i [2] and Fastsimcoal [3] suggested the existence of two historical refugia one located in the South of the distribution range, another that would correspond to a refugia for the Thompson lineage which is strongly differentiated, based on RADseq and whole genome sequences. Inferences also suggested that the Thompson area would have diverged approximately ~100 to 130 kyA followed and became reconnected in postglacial time. Yet, levels of net sequence divergence and of genetic diversity as well as the presence of long runs of homozygosity (ROH) are compatible with a recent bottleneck and more recent divergence of this population from the rest of the dataset[1,4].

Moreover, SMC++ analysis in [4], suggests a more recent divergence (in the range 5-60kyA). Therefore we aimed at validating the results from our precedent work suggesting that i) populations have expanded from a single refugia in postglacial time and ii) the Thompson have been isolated in post glacial time and then undergone a secondary contact [1]. 

The major novelty of our modelling approach is to include the possibility for population size change (expansion, bottleneck) in the ancestral population, in addition to size change in the daughter populations. Indeed a recent simulation study suggested that secondary contact may falsely be inferred under a true model of continuous migration if ancestral population size change is ignored [5]. In addition, we took advantage of our outgroup information to build unfolded site frequency spectra, providing richer information than our previous inferences [1]. As previously, our models included the effect of linked selection and barriers to gene flow which are respectively modeled through variation in effective population size and variation in effective migration rate. These models were applied to the exact same set of RADseq data [1], but based on a new unfolded joint site frequency spectrum constructed after remapping all data to the V2 genome. All unfolded jSFS were generated with ANGSD [6]. Model choice was performed using AICc.

We fitted four alternative models of divergence, described in [1] using ∂a∂i [2]:

1) Strict Isolation (SI)

2) Divergence with Ancient Migration (AM)

3) Isolation with Migration (IM)

4) Divergence followed by Secondary Contact (SC).

These models integrate the confounding effect of linked selection modeled as a reduction in effective population along the genome (suffix 2N) as well as barriers to gene flow reducing the local migration rate along the genome (suffix 2M).

As previously we compared the following major genetic group by fitting pairwise models of populations divergence for 5 pairs of populations:

* California vs Thompson

* Cascadia vs Thompson

* Alaska vs Thompson

* California vs Alaska

* Cascadia vs Alaska

Results

Model choice performed through AIC and ΔAIC revealed support for the model of ancient migration (AM) in all pairwise comparisons (Table S08, Table S09). In 3 out of the 5 comparisons, models that integrated population size change in the ancestral population received higher support. Models with either linked selection (suffix 2N) or barriers to gene flow (suffix 2M) were always supported compared to models that ignore these effects. Importantly, these models provided a better fit to the data than our previous work in terms of residual as shown in the figure below. Suggesting that our new inferences should be more reliable both in terms of model choice and parameter estimation. Parameter estimates are provided in Table S09. Our main parameter of interest here, the split time revealed that the populations have diverged between 5KyA and 45 KyA, which is more recent than our previous work. This result is more in line with SMC++ based inference (Fig S03) despite the very different modelling assumption of the two approaches.

In summary, our results do not support the existence of a separate refuge followed by secondary contact, but rather support a recent divergence with initial gene flow.

Figure Supp Note S1: Model and Residual for each pairwise comparison obtained from ∂a∂i. Each plot displays the observed jSFS (data), the modeled jSFS (model) and the residuals. The best model was inferred using ΔAIC and AIC weights (Table S2).
