# Supplementary material for: Allele surfing causes maladaptation in a Pacific salmon of conservation concern
Source: PLoS Genet. 2023 Sep 8;19(9):e1010918. doi: 10.1371/journal.pgen.1010918 (PMC10545117; doi:10.1371/journal.pgen.1010918)
Supplement: Supplementary file 15 [file pgen.1010918.s011.odt]

Supplementary Note S2: Relationship between GC3 and recombination

Instead of directly testing the association between πN/πS and recombination, we assessed the relationship between πN/πS and GC content at the third coding position (GC3). There were four reasons for that. First, population genetic estimates relies on the compound metric 4Ne*rho, rather than rho directly; so that strong variation in effective population size is a first source of bias. Second, gene flow is known to distort these results [7]. Third, under some conditions of mutation rates or low recombination, these methods may be biased [8]. Fourth, Ldhat and several related methods often assumed demographic equilibrium, a hypothesis that was strongly violated at the expansion front or in the Salmon river, showing signs of bottleneck and inbreeding.

Indeed, during the recombination process, GC biased gene conversion occurs (gBGC): during recombination, heteroduplexes mispairing are preferentially repaired in favor of G-C based, rather than A-T, this is the process of gGBC. The higher the recombination is, the higher the probability of errors is, and therefore an increase in GC content is to be expected, as observed in several species [9–16].

Unlike population scale estimates of recombination, GC3 estimates do not rely on a particular population genetic model that assumes demographic equilibrium, it should therefore not be biased by the process mentioned above (gene flow, variation in population size). It can be directly estimated from the data and the accuracy of measuring GC3 should not be particularly affected by variation in mutation or recombination rates. Finally, the GC3 should remain a good proxy although ideally, we should use GC3 at silent sites (GC3s) that are even less affected by selection [13], or 4-fold degenerated GC3.
