# Supplementary material for: Allele surfing causes maladaptation in a Pacific salmon of conservation concern
Source: PLoS Genet. 2023 Sep 8;19(9):e1010918. doi: 10.1371/journal.pgen.1010918 (PMC10545117; doi:10.1371/journal.pgen.1010918)
Supplement: Supplementary file 16 [file pgen.1010918.s012.odt]

Supplementary Note S3 Comparison of Provean and non-synonymous results.

Provean [17] measures the effect of a variation (v) on a protein A by measuring the similarity (using blast) of the protein A to a protein B before and after the introduction of the variation. The assumption is that a variation that reduces the similarity of protein A to the functional homolog protein B is more likely to cause a damaging effect. To this end, a change in the “alignment score” (delta score) can be used as a measure of change in “similarity” caused by a variation. A low delta score is interpreted as a deleterious effect of the variation, with a score below -2.5 being the recommended value to provide a tradeoff between specificity (i.e. a high confidence in the variants being identified as deleterious) and sensitivity (i.e. identifying a high number of deleterious variants). A high delta score is interpreted as neutral. The top 30 clusters of closely related proteins are used to compute a delta score, which are then averaged across clusters [17]

While we previously used Provean to identify potentially deleterious mutations, we argue here that such an approach, based on the protein conservation across many species, is similar in spirit to approaches such as GERPP score [18], whose population genetic properties have been well studied [19]. In particular, a major limit of these approaches is associated with their assumption of conservation across all the clades investigated, so that only mutation that was selected across all the studied clades will produce a significant score. Change in selection at a given site through time reduces the power to detect selection in our focal species. Moreover, these approaches were mostly designed for humans and mammals. Still, to verify the consistency of our results we run Provean on a random set of non-synonymous gene. The results provided in S15 Fig remained broadly consistent with those obtained from LoF and missense mutations in Figure 3.
